# Pomegranate By-Products as Natural Preservative to Prolong the Shelf Life of Breaded Cod Stick

**DOI:** 10.3390/molecules26082385

**Published:** 2021-04-20

**Authors:** Olimpia Panza, Amalia Conte, Matteo Alessandro Del Nobile

**Affiliations:** Department of Agricultural Sciences, Food and Environment, University of Foggia, Via Napoli, 25, 71122 Foggia, Italy; olimpia.panza@unifg.it (O.P.); matteo.delnobile@unifg.it (M.A.D.N.)

**Keywords:** pomegranate, byproducts, ready-to-cook fish, fortified food, sustainable food

## Abstract

This work evaluated the efficacy of pomegranate byproducts, specifically peel powder, as valid preservatives for food quality. Ready-to-cook cod sticks breaded with pomegranate peel powder were prepared. Shelf-life tests were conducted on breaded cod sticks during refrigerated storage (17 days) at 4 °C, monitoring the pH, microbiological and sensory quality. In addition, the nutritional quality of both the breaded and control samples was assessed. The results highlighted that active samples showed higher phenol and flavonoid content and higher antioxidant activity compared to the control fish, suggesting that pomegranate peel powder was responsible for a significant increase in cod stick nutritional quality. Furthermore, the cod stick active breading led to a delay in microbial growth without affecting the sensory properties; rather, it helped slow down the sensory attribute decline during the refrigerated storage. The data suggest that using pomegranate byproducts in breaded cod stick was effective in prolonging its shelf life, as well as improving its nutritional quality. Therefore, pomegranate peel powder can be considered as a potential resource as natural food preservative.

## 1. Introduction

The growing interest to extend the shelf life of fresh fish has led to numerous studies on the optimization of handling, refrigeration and transport, packaging practices and nonthermal methods to maintain the high quality and product safety of fish goods [1]. Fresh fish can easily deteriorate after being caught, due to endogenous enzymes and rapid microbial growth naturally present in fish or coming from surface contamination [2]. Moreover, changes in composition during fish decay can lead to protein degradation and lipid oxidation, as well as to changes in odor, flavor and texture, with a consequent unacceptability of fresh fish products within a few days. Therefore, it is inevitable to search novel prevention methods to extend fish shelf lives [1]. Numerous studies are currently focused on using natural ingredients to enhance the food quality and shelf life and to meet consumer demands for safer foods to avoid the use of synthetic preservatives [3,4]. In particular, modern consumers are increasingly looking for healthy and sustainable products [5]. Therefore, the idea of replacing the practice of synthetic preservatives as sorbic acid and sorbates with plant-based compounds is of high interest, especially if extracted from cheap, abundant and sustainable agricultural sources, as byproducts [6,7,8]. Generally, fruit and vegetable byproducts are the most abundant materials among food byproducts, accounting for about 10–35% of the raw mass [9,10,11]. These byproducts have enormous potential to be recycled, being rich in polyphenols and flavonoids and, thus, playing an important role as both antioxidant and antimicrobial agents [12,13]. Many studies demonstrate that agricultural byproducts are useful for human nutrition thanks to their antioxidant and antiviral properties [14,15,16]. A recent publication of Dilucia et al. [17] gave insight on the possibility of exploring the potential of fruit and vegetable byproducts also for food packaging applications.

Food applications and potential health benefits of pomegranate and its derivatives have been abundantly discussed [18]. Li et al. [19] reported that pomegranate fruit is very rich in bioactive compounds. Similar results were reported by Akhtar et al. [20] and Orak et al. [21] and, more recently, by Derakhshan et al. [22], who confirmed a positive correlation between the presence of phenols and flavonoids and fruit antioxidant activity. Several studies have also shown that higher bioactive compounds are concentrated in the peel rather than in other parts of the fruit [20,23,24,25]. In particular, pomegranate peel comprises nearly a 30–40% portion of the fruit. It is rich source of tannins and other phenolic and flavonoid compounds, thus having a higher antioxidant capacity than seeds and pulp [26]. Pomegranate fruit and its derivatives were effective in retarding the process of lipid oxidation by both in vitro and in vivo assays [6,7,8]. Furthermore, the pomegranate peel extract has antimicrobial and antifungal properties, therefore playing a dual role [23,27] that can also be affected by the cultivar [28]. These results suggest that the peel can be an effective and natural option for synthetic preservative agents [7,18]. For these reasons, it is interesting to investigate at which extent a pomegranate peel can preserve food quality during storage [29]. To the best of our knowledge, the scientific literature up to now only focused its attention on the potential of pomegranate juice, peel and seed extracts [30,31,32,33,34], and no information has been reported on the effects of the peel, as it is, on fresh food shelf lives, thus avoiding any preliminary extraction process.

In this context, this study tested pomegranate peel powder on breaded cod sticks. To this aim, the breading was prepared with and without adding pomegranate peel powder. During refrigerated storage, vital cell loads, pH and sensory quality were monitored to assess whether the addition of the active ingredient to the classic breading could extend the shelf life of the packaged food product. In addition, differences in phenolic compounds, flavonoids and antioxidant activity between the control and the active samples were also assessed to evaluate the nutritional quality of the tested samples.

## 2. Results and Discussion

### 2.1. Total Phenols, Total Flavonoids, and Antioxidant Activity of Breaded Cod Sticks

The nutritional quality of breaded cod sticks added with pomegranate peel powder was evaluated in terms of total phenols (mg GAE/g dw), flavonoids (mg QE/g dw) and antioxidant activity (mg Trolox equivalent/g dw); the data obtained in this study are shown in Table 1. As can be seen, the nutritional quality of both raw samples (R-Con, R-A, R-B and R-C) and cooked samples (C-Con, C-A, C-B and C-C) was assessed. According to the data shown in Table 1, the addition of pomegranate peel powder in the breading of cod sticks significantly improved their nutritional quality. In particular, as long as the raw samples are concerned, the total phenol content was more than eight times higher in all fortified samples compared to the control; whereas, in the case of cooked samples, the total phenol content was found to be five times higher in all active samples compared to the control. A similar trend was found for flavonoids. In fact, the raw active samples showed a flavonoid concentration higher than the control fish (5.43, 5.80, 7.19 and 0.54-mg QE/g dw, respectively, for R-A, R-B, R-C and R-Con). After cooking, the flavonoids in active samples slightly decreased. Other findings in the scientific literature reported that the degradation of flavonoids is a combination of several mechanisms depending on the operating conditions (heating, mechanical and domestic processes) and food matrix [35]. Therefore, in some cases, cooking may increase their availability, while, in others, it may reduce their content, as shown by Dolinsky et al. [36] and Murador et al. [37]. However, the difference between active and control samples remained still significant (2.89, 2.19, 4.54 and 1.08-mg QE/g dw, respectively, for C-A, C-B, C-C and C-Con). As reported in the literature, phenol and flavonoid content are precursors of antioxidant capacity and can be used as a preliminary marker for any product to be used as natural source of antioxidants in functional foods [31]. As already mentioned, pomegranate peel has a higher antioxidant capacity than arils and seeds [20], thus making it a powerful source of bioactive compounds [26]. As shown in Table 1, a direct correlation between polyphenol content and antioxidant activity is clearly evident, confirming what was found by other authors. In fact, Kannat et al. [32] demonstrated that the incorporation of pomegranate peel extracts in ready-to-eat meat products resulted in a higher antioxidant and antimicrobial activity in the final product. Furthermore, Tarkhasi [31] showed how the use of pomegranate peel extract contributed positively to extend the shelf life of silver carp fillet by reducing the microbial load and increasing antioxidant activity, with a consequent reduction of lipid oxidation. Similar results were also obtained by Martinez at al. [33], who incorporated pomegranate extracts into fish patties with the aim to reduce lipid oxidation and microbial load. Finally, in the study of Incoronato et al. [29] focused on pancakes enriched with pomegranate byproducts, a significant increase in the nutritional quality of the product was found.

### 2.2. Microbial Quality of Breaded Cod Sticks and pH

**Figure 1:**
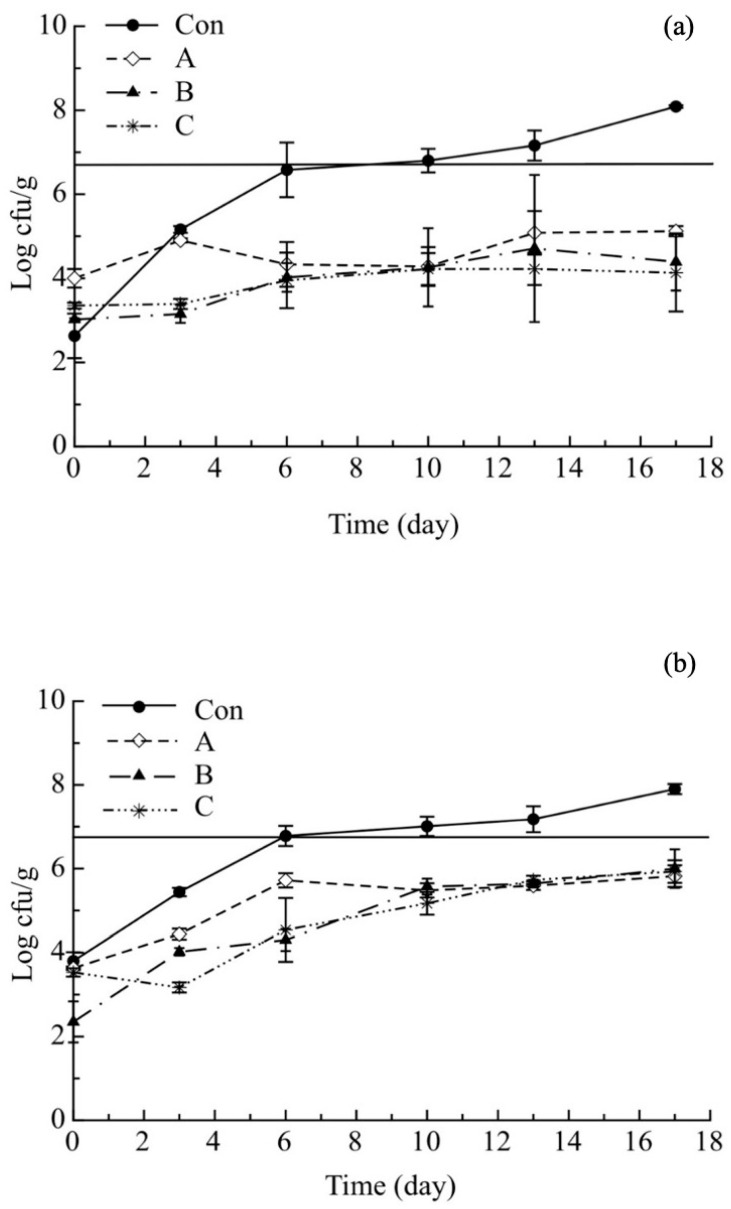
Evolution of total mesophilic (**a**) and psycrhrotrophic (**b**) bacteria in breaded cod sticks during storage at 4 °C. Data are presented as mean ± SD (*n* = 2). Symbols: experimental data; Solid Line: threshold for microbial acceptability set to 5 × 10^6^ log cfu/g. Con: sticks breaded with sole no active mix; A: sticks breaded with sole active mix; B: sticks breaded with pomegranate peel powder/no active mix; C: sticks breaded with pomegranate peel powder/active mix.

The microbial quality decay of the investigated breaded cod sticks was determined by monitoring the viable cell concentration of total mesophilic and psychrotrophic bacteria (TMB, TPB), *Pseudomonas* spp. (PSE), *Shewanella putrefaciens* (HSPB) and *Photobacterium phosphoreum* (PHAB). The TMB and TPB evolution of the investigated breaded cod sticks is shown in Figure 1a,b. During the 17 days of storage, the TMB viable cell concentration (Figure 1a) of the investigated samples gradually increased; however, the TMB of Ctrl sample increased faster and exceeded the microbial limit (set at 5 × 10^6^ cfu/g) after about 6 days of storage.

The active samples showed a slower increase in mesophilic counts compared to the control. In particular, at the end of the storage period A, B and C samples reached values of 5.12, 4.39 and 4.13 log(cfu/g), respectively; whereas the Con sample reached 8.09 log(cfu/g). These results suggest that pomegranate peel powder was effective in slowing down the microbial growth. A similar trend has also been observed on the psychrotrophic counts in all the investigated samples (Figure 1b). The results obtained for TMB and TPB are similar to those found in several studies where the antimicrobial effectiveness of pomegranate peel extract was evaluated. In particular, Tarkhasi [31], Kannat et al. [32] and Martinez et al. [33] used pomegranate peel extract to control microbial growth in fish or chicken-based products, and all of them found that the extract significantly contributed to control microbial stability. Data obtained in the current study prove that also pomegranate peel maintains its efficacy against spoiling bacteria. It is well-known that the deterioration of fish products could be ascribed to the presence of H_2_S off-odors and off-flavors, which in turn are produced by specific microbial groups [4]. For this reason, the microbial concentrations of *Pseudomonas* spp. (PSE), hydrogen sulphide-producing bacteria (HSPB—*Shewanella putrefaciens*) and psychrotolerant and heat labile aerobic bacteria (PHAB—*Photobacterium phosphoreum*) were also monitored.

**Figure 2:**
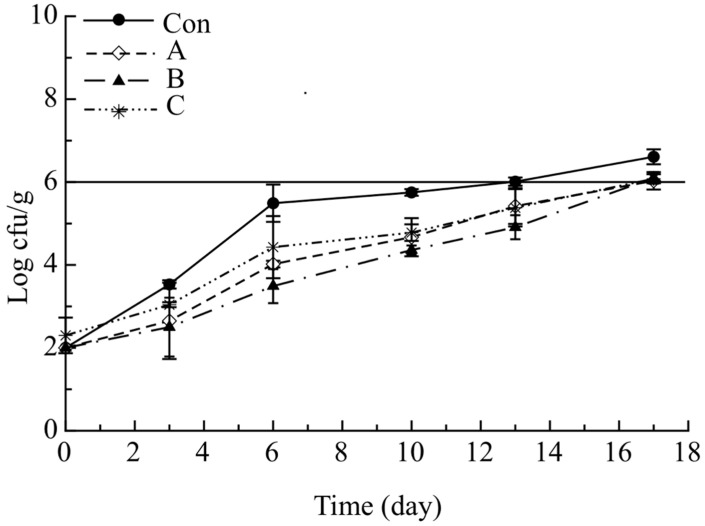
Evolution of *Pseudomonas* spp. in breaded cod sticks during storage at 4 °C. Data are presented as mean ± SD (*n* = 2). Symbols: experimental data; Solid Line: threshold for microbial acceptability set to 10^6^ log cfu/g. Con: sticks breaded with sole no active mix; A: sticks breaded with sole active mix; B: sticks breaded with pomegranate peel powder/no active mix; C: sticks breaded with pomegranate peel powder/active mix.

As can be observed from data shown in Figure 2, at the beginning of the storage period PSE counts of investigated cod stick samples were substantially similar, ranging from 2.0 to 2.3 log(cfu/g). During storage, a slower growth in the *Pseudomonas* spp. viable cell concentration of active samples was observed if compared to the control sample, which reached the microbiological acceptability limit (10^6^ cfu/g) on the ninth day, whereas all the active samples never reached the limit during the 17 days of monitoring.

The abovementioned results suggest that adding pomegranate peel powder delayed microbial growth. In fact, during the observation period a substantial difference between active samples and Con of about 1 log cycle was observed. Similar results were reported by Khan and Hanee [27], who showed that the bioactive compounds of pomegranate peel extract (polyphenols, tannins, flavonoids and anthocyanins) have antibacterial activity against the strains of *Escherichia coli*, *Pseudomonas aeruginosa* and *Staphylococcus aureus*.

**Figure 3:**
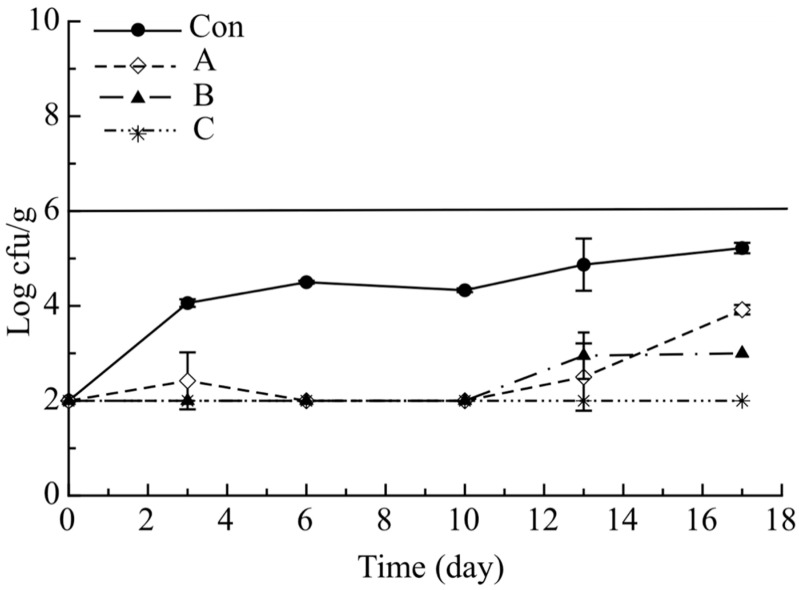
Evolution of *Shewanella* in the breaded cod sticks during storage at 4 °C. Data are presented as mean ± SD (*n* = 2). Symbols: experimental data; Solid Line: threshold for microbial acceptability set to 10^6^ log cfu/g. Con: sticks breaded with sole no active mix; A: sticks breaded with sole active mix; B: sticks breaded with pomegranate peel powder/no active mix; C: sticks breaded with pomegranate peel powder/active mix.

Figure 3 shows the evolution of *Shewanella* viable cell concentration in the investigated samples during refrigerated storage. A similarity can be observed between the evolution of *Shewanella* viable cell concentration and that of aerobic mesophilic bacteria. In particular, HSPB counts for all investigated samples were initially low, about 2.0 log (cfu/g), suggesting a good initial quality of cod fish. During refrigerated storage, a significant microbial growth inhibition was evident in all active samples compared to the control sample, even though during the observation period no samples exceed the microbiological acceptability limit (10^6^ cfu/g). A low viable cell concentration was also recorded for *P. phosphoreum*, where the cell load was always below the detection level for all investigated samples (data not shown). Instead, for *Enterobacteriaceae*, there was a substantial difference between control and all active samples. The Con sample began to increase from the third day of storage (3.46 log(cfu/g)), with a steady trend along the entire observation period (data not shown). To sum up, the control fish became unacceptable for excessive TMB and TPB proliferation around the sixth day of storage, whereas all the investigated active fish samples remained acceptable during the entire observation period (17 days) with cell loads that never reached microbial thresholds.

**Figure 4:**
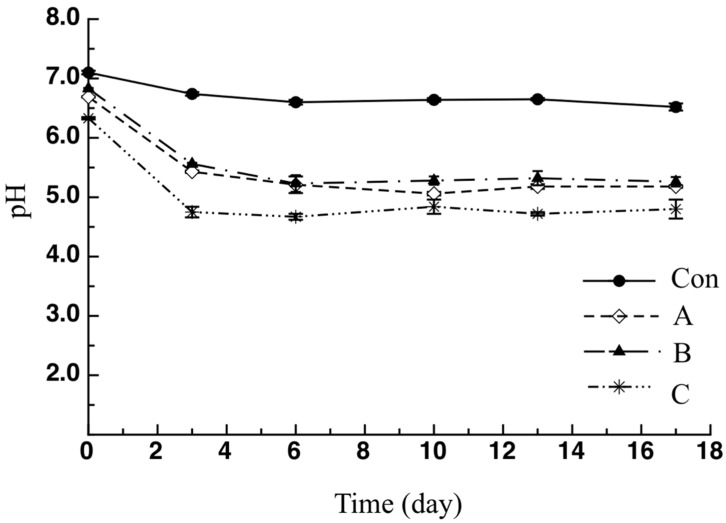
Trend of pH of breaded cod sticks. Data are presented as mean ± SD (*n* = 2). Symbols: experimental data; Con: sticks breaded with sole no active mix; A: sticks breaded with sole active mix; B: sticks breaded with pomegranate peel powder/no active mix; C: sticks breaded with pomegranate peel powder/active mix.

Another important physical-chemical characteristic is the pH, used to establish the quality and durability of a given food product. The pH evolution during refrigerated storage of the investigated samples is reported in Figure 4. As shown, the initial pH value of the control sample was 7.10 ± 0.03, whereas it was 6.69 ± 0.01, 6.83 ± 0.01 and 6.33 ± 0.03 in the A, B and C samples, respectively. Similar results for the initial pH of fish-based foods were also reported by Tarkhasi [31]. From the data shown in the figure, it can be observed that in the first days of storage the addition of pomegranate peel powder reduced the pH compared to the control sample, suggesting a direct effect related to the acid pH of the pomegranate peel powder, as also observed by Ullah et al. [23]. Subsequently, the pH of the active samples remained substantially unchanged for the entire storage period. A similar trend was found in the study of Incoronato et al. [29].

### 2.3. Sensory Quality of Breaded Cod Sticks

**Figure 5:**
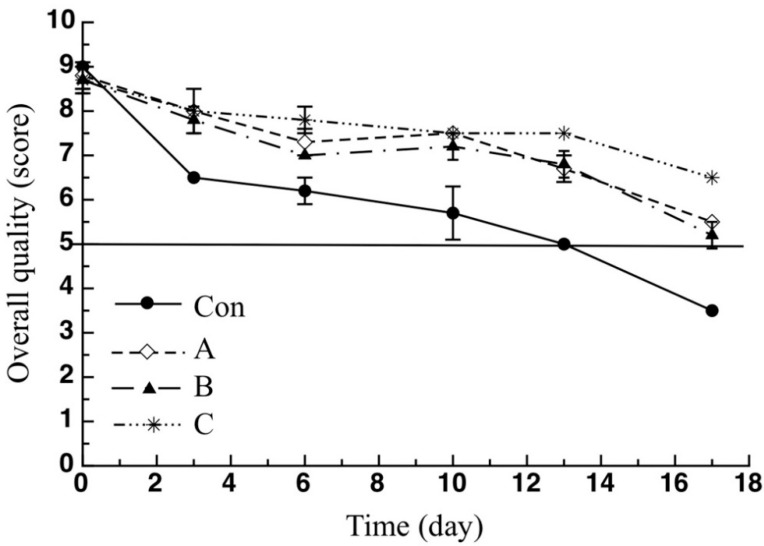
Overall quality of the breaded cod sticks during storage. Data are presented as mean ± SD (*n* = 2). Symbols: experimental data; Solid Line: threshold for sensory acceptability set to 5. Con: sticks breaded with sole no active mix; A: sticks breaded with sole active mix; B: sticks breaded with pomegranate peel powder/no active mix; C: sticks breaded with pomegranate peel powder/active mix.

**Figure 6:**
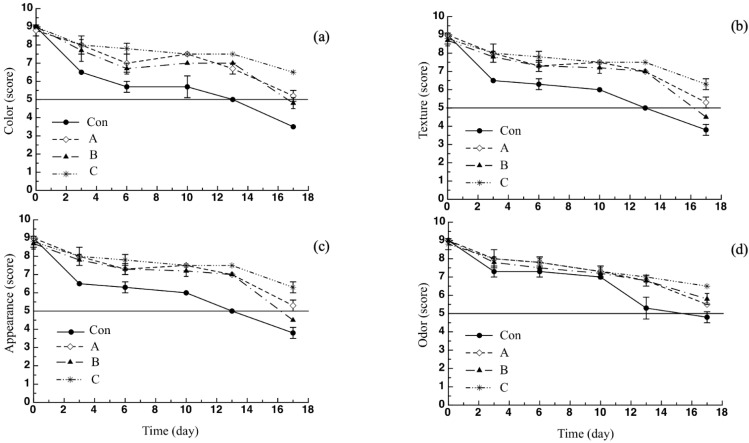
Sensory attributes of breaded cod sticks: (**a**) color, (**b**) texture, (**c**) appearance, (**d**) odor. Data are presented as mean ± SD (*n* = 2). Symbols: experimental data; Solid Line: threshold for sensory acceptability set to 5. Con: sticks breaded with sole no active mix; A: sticks breaded with sole active mix; B: sticks breaded with pomegranate peel powder/no active mix; C: sticks breaded with pomegranate peel powder/active mix.

As reported beforehand, a panel of expert judges was used to assess the breaded cod stick sensory properties in terms of odor, color, texture and general appearance. The mean values of sample overall quality were reported in Figure 5. As can be seen, during the 17 days of observation the control sample reached the acceptability threshold (score = 5) after 11 days, whereas the overall quality of all active samples was above the acceptability limit up to the end of the observation period. It is worth noting that sample C overall quality was always higher than that of the other two active samples. In particular, at the end of the observation period, the overall quality of sample C was well above the acceptability threshold (6.3), whereas that of samples A and B were much closer to the abovementioned limit at 5.5 and 5, respectively. Details about the trends of each single sensory attribute are reported in Figure 6. Specifically, for the control sample, a marked decline of the color (Figure 6a) was observed after 3 days (6.5) compared with the active samples of A (8.0), B (7.7) and C (8.0), which recorded much higher scores. In fact, the control sample was considered to be much darker if compared to the active sticks. A similar trend was found in terms of sample texture (Figure 6b) and appearance (Figure 6c). In fact, the Con sample was considered less compact and more humid than the active samples. As far as odor is concerned (Figure 6d), all samples recorded a score higher than the acceptability threshold without substantial differences among them until the 13th day, after which, there was a faster decay of this specific sensory attribute in the case of the control sample compared to the active fish. At the end of the storage period, a value of 4.8 was observed for the control sample, whereas the samples A, B and C obtained scores of 5.5, 5.8 and 6.5, respectively.

The latter result could be related to the growth of off-odor produced by microbial proliferation. In fact, as discussed beforehand, pomegranate peel powder has been proven to be effective in slowing down the growth of microorganisms responsible for off-odors development. A similar trend was also found in the study by Khodanazar [34], where a faster decline in sensory attributes and, in particular, in the odor, directly related to microbial growth, was observed for untreated fish fillets compared to samples treated with pomegranate extract. As can clearly be inferred from what reported beforehand, the addition of pomegranate peel powder to cod stick breading does not negatively affect the final product sensory quality; rather, it contributes to improving it, slowing down its decay during refrigerated storage. Thus, the control fish remained acceptable for about 11 days, whereas all the investigated active sticks were found acceptable until the end of the monitoring period.

## 3. Materials and Methods

### 3.1. Raw Materials

Refrigerated salted cod fillets and pomegranate fruits were purchased at local market (Foggia, Italy). Pomegranates were carefully washed with tap water to remove residues, immersed for 1 min in chlorinated water (20 mL L^−1^) and rinsed. The pomegranates were cut manually to separate the seeds/arils, the pulp and the peel. Pomegranate juice was extracted using a fruit extractor (Delonghi, Italy) and stored at −18 °C until it was used. Pomegranate peel and pulp were separated, cut into small pieces with a sharp knife and dried in a food dehydrator (Melchioni-Babele, Milan, Italy) at 38 °C for 48 h. The dried product was reduced to powder in a laboratory blender and then sieved using a 500-μm sieve. Fine peel and pulp powders were stored separately in plastic bags at 4 °C in an environment protected from light. Other ingredients used to prepare the cod stick breaded fish, such as breading, spices, potato flakes and fresh milk, were purchased at a local market (Foggia, Italy).

### 3.2. Breaded Cod Stick Preparation

Cod fillets were coarsely desalted, soaked and stored at 4 ± 1 °C for five days, changing the water every day. On the sixth day, cod fillets were drained to remove excess water for about half an hour, and the skin was removed. Then, fillets (about 67% *w*/*w* water and about one percent *w*/*w* NaCl) were cut into sticks of about 12 g. Two mixtures were prepared: no-active mixture (no-active mix) containing breading with fish spices and potato flakes in a ratio of 1:1 and active mixture (active mix) prepared with pomegranate peel powder and non-active mix in a ratio of 1:1. Four sample types were prepared (i.e., Con, A, B and C). The control sample (Ctrl) was obtained as follows: after dipping in a solution of water and milk (1:1), the sample was breaded in the no-active mix by repeating the passage twice; then, it was manually compacted, placed above a food tray with a pad and packaged in air using a high-barrier film bag (multilayer film Nylon/Polyethylene) with a thickness of 150 µm, provided by Biochemia (Bari, Italy) and kept under refrigeration (4 ± 1 °C). All active samples were prepared using the same procedure as for the control sample but using different breading: active sample A (A) was breaded twice in the active mix, active sample B (B) was first breaded with pomegranate peel powder and then with the no-active mix and active sample C (C) was first breaded with pomegranate peel powder and then with the active mix. All the samples were stored at 4 ± 1 °C for 17 days. Uncooked samples were used to determine the pH, sensory and microbiological quality during the entire storage period. The nutritional quality of both cooked and uncooked samples was also assessed. Samples were cooked at 200 °C for 15 min in an electric oven (Europa Forni, Vicenza, Italy). Two replicates for each treatment were evaluated to ensure repeatability.

### 3.3. Chemicals

Folin–Ciocalteu reagent, gallic acid monohydrate, ethanol, ABTS (2,2-azino-bis(3-ethylbenzothiazoline-6-sulfonic acid)diammonium salt), potassium persulfate (K_2_S_2_O_8_), Trolox (6-hydroxy-2,5,7,8-tetramethylchroman-2-carboxylic acid), aluminum chloride (AlCl_3_), sodium nitrite (NaNO_2_), sodium hydroxide solution (NaOH) and quercetin were supplied from Sigma-Aldrich (Milan, Italy). Anhydrous sodium carbonate (Na_2_CO_3_) was supplied from Carlo Erba (Milan, Italy). All reagents were of analytical grade.

### 3.4. Extraction of Bioactive Compounds

For chemical analyses, both raw (R-Con, R-A, R-B and R-C) and cooked samples (C-Con, C-A, C-B and C-C) were first subjected to drying at 35 °C in a ventilated stove (BINDER GmbH, Tuttlingen, Germany), milled to obtain a powder and then subjected to extraction, as reported by Cedola et al. [38]. Briefly, 1 g of dried sample was mixed with 20 mL of equal mixture water:ethanol (*v*/*v*) in Erlenmeyer flasks and maintained for 30 min in a water bath (GRANT OLS200, Cambridge, UK) at 60 °C under agitation. The extracts were filtered to obtain clear supernatants. Triplicate extractions were made for each sample.

### 3.5. Determination of Total Phenols Content, Total Flavonoids and Antioxidant Activity

All the chemical analyses were performed the same day the samples were prepared. Total phenols were determined according to the Folin–Ciocalteu method, as reported by Cedola et al. [38]. The colorimetric method allowed to quantify the total phenol content as milligrams of gallic acid equivalents (GAE)/gram of dry weight (dw), according to a calibration curve (3.12–100 mg/L; R^2^ = 0.9934). Total flavonoid content was determined using the aluminum chloride colorimetric method, according to Cedola et al. [38]. The measure was conducted at 415 nm with a spectrophotometer (UV1800; Shimadzu Italia S.R.L; Milan, Italy) and total flavonoids were expressed as milligrams of quercetin equivalent (QE)/gram of dry weight (dw). Quercetin standard solutions were used for constructing the calibration curve (6.25–500 mg/L; R^2^ = 0.994). The antioxidant activity of breaded cod sticks was assessed using the ABTS (2,2-azino-bis (3-ethylbenzothiazoline-6-sulfonic acid diammonium salt) assay method. The test is based on the ability of the antioxidants to interact with the radical cation 2,2′-azino-bis(3-ethylbenzothiazoline-6-sulfonic acid) (ABTS+), inhibiting its absorption at 734 nm. The analysis was conducted according to the method used by Cedola et al. [38]. A calibration curve was built using Trolox as the standard at concentrations between 3.25 mg/L and 600 mg/L (R^2^ = 0.9976), and the antioxidant activity was expressed as milligrams of Trolox equivalents/gram of dry weight (dw). All tests were conducted in triplicate.

### 3.6. Microbiological Analyses and pH Determination

Control and active samples (20 g each) were aseptically weighed into a sterile stomacher bag, diluted with peptone water (dilution 1:10) and homogenized for 90 s with a Stomacher LAB Blender 400 (Pbi International, Milan, Italy). Serial dilutions were plated onto specific media in Petri dishes to enumerate *Pseudomonas* spp., hydrogen sulfide-producing bacteria (HSPB), psychrotolerant and heat-labile aerobic bacteria (PHAB) and mesophilic and psychrotrophic bacteria, Enterobacteriaceae, according to Danza et al. [39]. The conditions used for counting HSPB and PHAB were suggested by the Nordic Committee on Food Analyses (2006). All media and supplements were obtained from Oxoid (Milan, Italy). The microbiological analyses were conducted twice on two samples. Results are expressed as log cfu/g. Microbial thresholds were set to 5 × 10^6^ cfu/g for total viable mesophilic and psychrotrophic bacteria, 10^6^ cfu/g for *Pseudomonas* spp. and *Shewanella* 10^7^ cfu/g for *Photobacterium* [40].

The measurement of pH was performed in triplicate on the first homogenized dilution of fish samples, using a pH meter (Crison, Barcelona, Spain). Two samples were used for each measurement. Microbiological analyses and pH were analyzed at the initial time and after 3, 6, 10, 13, and 17 days of refrigerated storage at 4 °C.

### 3.7. Sensory Analysis

The quantitative descriptive analysis (QDA) was used for a sample comparison, according to the guidelines of the Codex Alimentarius Commission. To this aim, breaded cod sticks were submitted to a panel of five trained judges. The panelists had familiar eating habits with fish and fish products and had experience in the evaluation of burgers and fillets based on fish. They were retrained for two days (2-h sessions) to establish the appropriate attributes for sensory evaluation and to minimize individual differences and ensure repeatability of the results. The panelists were asked to give judgments on odor, color, appearance, texture, and overall quality using a nine-point scale. In the scale, 9 corresponded to excellent, 8 to very good, 7 to good, 6 to reasonable, 5 to not good (acceptable limit), 4 to dislike, 3 to bad, 2 to very bad and 1 to completely unacceptable [41]. Before the sensory analysis, samples were sliced with a knife without removing the breading crust. Samples were differently coded and presented to each panelist simultaneously in random order.

### 3.8. Statistical Analysis

Experimental data were compared by one-way analysis of variance (ANOVA). A Duncan’s multiple range test, with the option of homogeneous groups (*p* < 0.05), was performed to determine significant differences among the samples. STATISTICA 7.1 for Windows (StatSoft, Inc., Tulsa, OK, USA) was used.

## 4. Conclusions

In this work, pomegranate byproducts were included in cod stick breading to improve the final product quality from a nutritional perspective, as well as to prolong its shelf life by about three times. The obtained results showed also a significant improvement in the nutritional quality of fortified samples after cooking. In fact, final cooked cod sticks breaded with pomegranate peel powder were characterized by phenolic compounds from three to five-fold higher than the cooked control, flavonoids from two to seven-fold higher than the cooked control and antioxidant activity from two to four-fold higher than the cooked control fish. Furthermore, the results underlined that the addition of pomegranate peel powder also slowed down the microbial growth during refrigerated storage, without negatively altering its sensory characteristics. Rather, as deduced from the results found, the addition of pomegranate peel powder to the cod stick breading considerably reduced the sensory decline during refrigerated storage compared to the control sample. Therefore, it can be concluded that the reuse of pomegranate by-products in fresh food industry could be a sustainable way to reduce environmental impact and costs associated with byproducts disposal, with great advantages to the product quality and its shelf life.

## Figures and Tables

**Table 1:** Total phenols, total flavonoids and antioxidant activity of raw and cooked cod sticks.

Samples	Total Phenols (mg GAE/g dw) ± SD	Total Flavonoids (mg QE/g dw) ± SD	Antioxidant Activity (mg Trolox/g dw) ± SD
R-Con	1.17 ± 0.03 ^a.A^	0.54 ± 0.16 ^a.A^	1.05 ± 0.34 ^a.A^
R-A	8.13 ± 0.74 ^b.A^	5.43 ± 0.55 ^b.A^	5.15 ± 0.12 ^b.A^
R-B	8.44 ± 2.05 ^b.A^	5.80 ± 1.53 ^b.A^	5.47 ± 0.49 ^b.c.A^
R-C	10.59 ± 2.89 ^b.A^	7.19 ± 2.11 ^b.A^	7.46 ± 2.18 ^c.A^
C-Con	1.24 ± 0.12 ^a.A^	0.74 ± 0.35 ^a.A^	1.08 ± 0.19 ^a.A^
C-A	5.08 ± 2.31 ^a.b.A^	2.87 ± 0.77 ^a.b.B^	2.89 ± 0.67 ^a.b.B^
C-B	4.19 ± 0.72 ^b.B^	1.66 ± 0.58 ^b.B^	2.19 ± 0.94 ^b.B^
C-C	6.19 ± 2.18 ^b.A^	5.8 ± 0.81 ^c.A^	4.54 ± 0.96 ^c.A^

Data are presented as mean ± SD (*n* = 3). Data in each column with different superscript lowercase letters (a–c) show significant differences between raw control and active samples (R-Con and R-A, R-B and R-C) and between cooked control and active samples (C-Con and C-A, C-B and C-C), while different superscript capital letters (A–B) show significant differences between each raw and cooked sample (R-Con and C-Con; R-A and C-A; R-B and C-B; R-C and C-C) (*p* < 0.05). GAE = gallic acid equivalents; QE = quercetin equivalent; R-Con: raw sticks breaded with sole no active mix; R-A: raw sticks breaded with sole active mix; R-B: raw sticks breaded with pomegranate peel powder/no active mix; R-C: raw sticks breaded with pomegranate peel powder/active mix; C-Con: cooked sticks breaded with sole no active mix; C-A: cooked sticks breaded with sole active mix; C-B: cooked sticks breaded with pomegranate peel powder/no active mix; C-C: cooked sticks breaded with pomegranate peel powder/active mix.

## Data Availability

The raw data will be made available upon request.

## Sample Availability

Samples of the compounds are not available from the authors.
